# The relation of work-related factors with ambulatory blood pressure and nocturnal blood pressure dipping among aging workers

**DOI:** 10.1007/s00420-019-01510-8

**Published:** 2019-12-31

**Authors:** Saana Karelius, Jussi Vahtera, Jaana Pentti, Annika S. Lindroos, Pekka Jousilahti, Olli J. Heinonen, Sari Stenholm, Teemu J. Niiranen

**Affiliations:** 1 Department of Internal Medicine, University of Turku, Turku, Finland; 2 Department of Public Health, University of Turku, Turku, Finland; 3 Centre for Population Health Research, University of Turku and Turku University Hospital, Turku, Finland; 4 Clinicum, Faculty of Medicine, University of Helsinki, Helsinki, Finland; 5 Department of Public Health Solutions, National Institute for Health and Welfare, Helsinki, Finland; 6 Paavo Nurmi Centre & Department of Health and Physical Activity, University of Turku, Turku, Finland; 7 Division of Medicine, Turku University Hospital, Turku, Finland

**Keywords:** Job control, Job demand, Job strain, Occupational status, Shift work, Work-time mode

## Abstract

**Objectives:**

Individuals with reduced nocturnal blood pressure (BP) dipping are at increased risk of cardiovascular disease compared to persons with normal BP dipping. Although the relation of work-related factors and BP has been studied extensively, very little is known of the association between work-related factors and 24-h BP patterns in aging workers. We examined the cross-sectional relation of work-related risk factors, including occupational status, work-time mode, job demands and job control, with ambulatory BP in aging workers, focusing on nocturnal BP dipping.

**Methods:**

208 workers (mean age 62 ± 3 years; 75% women) from two Finnish population-based cohort studies underwent 24-h ambulatory BP monitoring. Work-related factors were inquired using a questionnaire. Nocturnal BP dipping was calculated as [1 − (asleep BP/awake BP)] × 100.

**Results:**

Shift workers demonstrated a higher nocturnal diastolic BP dipping than regular day workers (19% vs. 17%, *p* = 0.03) and had a significantly higher systolic awake BP than regular day workers (136.5 mmHg vs. 132.5 mmHg, *p* = 0.03). Participants with high job demands demonstrated a smaller nocturnal systolic BP dipping than participants with low job demands (14% vs. 16%, *p* = 0.04). We did not observe significant differences in nocturnal systolic or diastolic BP dipping between groups categorized by occupational status or job control.

**Conclusions:**

Although shift workers have a higher daytime BP than regular daytime workers, they exhibit greater nighttime BP dipping. Participants with high job demand had smaller nighttime BP dipping than participants with low job demand. Job control or occupation did not affect the 24-h ambulatory BP profile of aging workers.

## Introduction

High blood pressure (BP) is one of the leading risk factors for premature death worldwide (GBD 2015 Risk Factors Collaborators 2016). In addition to traditional risk factors for hypertension (e.g., obesity and high salt intake) (GBD 2015 Risk Factors Collaborators 2016; He et al. 2013), work-related factors, such as job strain and shift work, have been found to associate with hypertension (Esquirol et al. 2011; Gilbert-Ouimet et al. 2014).

After the introduction of the job demands–control model of occupational stress by Karasek in the 1970s, there has been growing interest to study the relation of job strain (i.e., imbalance between job demands and job control) and susceptibility to illness. Job strain is known to associate with elevated office BP, but this association may be even stronger when BP is measured using 24-h ambulatory monitoring (Gilbert-Ouimet et al. 2014). Compared to office BP, ambulatory BP offers possibility to measure nocturnal BP, a particularly strong predictor of cardiovascular morbidity and mortality (Hermida et al. 2011; Kikuya et al. 2005). Furthermore, the nocturnal decline in BP (i.e., nocturnal BP dipping) has been shown to have incremental prognostic value on cardiovascular events independent of 24-h systolic BP, particularly among hypertensive patients (Salles et al. 2016). In previous studies, individuals with reduced nocturnal BP dipping (non-dippers) have been noted to have an increased risk for cardiovascular diseases compared to persons with normal nocturnal BP dipping (dippers) (Salles et al. 2016).

Prior studies on the association between job strain and ambulatory BP have discovered that higher job strain is positively associated with systolic BP and to a lesser extent with diastolic BP (Cesana et al. 1996; Clays et al. 2007; Fauvel et al. 2001, 2003; Landsbergis et al. 1994, 2003a, b; Maina et al. 2011; Schnall et al. 1992, 1998; Tobe et al. 2005, 2007). However, it is unclear how job demands and job control separately associate with ambulatory BP and especially with nocturnal BP dipping in aging workers (Landsbergis et al. 2013). Two previous studies have examined the relation of job strain and nocturnal BP dipping in middle-aged participants (Clays et al. 2011; Fan et al. 2013), and only one study (Mc Carthy et al. 2014) with job control as the only exposure variable has focused on nocturnal BP dipping in the older workers (aged 50–69 years).

Another important work characteristic in terms of 24-h BP is work-time mode. Shift work is associated with circadian misalignment (Douma and Gumz 2018) which can affect BP profiles (Morris et al. 2017). Indeed, it has been shown that shift work raises BP and shift workers are classified more often as non-dippers than day workers (Fialho et al. 2006; Kitamura et al. 2002; Lo et al. 2008; Munakata et al. 2001; Ohira et al. 2000; Yamasaki et al. 1998). Again, most of these studies included only individuals aged under 40 years or had a study sample of under 60 participants. It is, therefore, unclear whether the association between work-related factors and diurnal BP profile persists among aging workers who are at a considerably greater absolute risk of cardiovascular disease than younger individuals.

The aim of this study was to examine the association of work-related factors, including occupational status, work-time mode, job demands, and job control with ambulatory BP patterns among aging workers (age ≥ 55 years). We particularly focused on the association of work-related factors with nocturnal BP dipping (defined as BP decrease during sleeping time) which has been scarcely studied previously.

## Methods

### Study sample

The study sample consisted of participants from two population-based cohort studies: the Finnish Retirement and Aging Study (FIREA) (Leskinen et al. 2018a) and the Dietary, Lifestyle, and Genetic determinants of Obesity and Metabolic syndrome study (DILGOM) (Lindroos et al. 2016).

FIREA is a longitudinal cohort study of older adults in Finland established in 2013 (Leskinen et al. 2018a). The eligible population for the FIREA study cohort included all public sector employees with an estimated retirement date between 2014 and 2019 who worked in 2012 in Southwest Finland or in the nine selected cities or five hospital districts around Finland and who responded to at least one of the FIREA questionnaires (*N* = 6679). After responding to the questionnaire, Finnish-speaking participants with an estimated retirement date between 2017 and 2019, who lived in the Southwest Finland and were still working, were invited to participate in the clinical sub-study (*n* = 773). Of them, 290 participated in the study between September 2015 and May 2018. We excluded 115 persons for not undergoing ambulatory BP measurements (due to refusal to participate, unavailable cuff size, arrhythmias or markedly elevated BP (182/101 mmHg in *n* = 1 FIREA participant)), 14 individuals for an insufficient number of valid ambulatory BP measurements (< 20 awake measurements or < 7 asleep measurements) (Parati et al. 2014) and 12 persons who had missing data on covariates. Thus, 149 FIREA participants were included in the analyses.

A second study sample was drawn from the participants of Dietary, Lifestyle, and Genetic determinants of Obesity and Metabolic syndrome (DILGOM) study that included a random sample of individuals living in Southwest Finland (Lindroos et al. 2016). The DILGOM study was originally carried out in 2007, and of the participating individuals, 493 were also included in the cardiovascular sub-study. In 2014, of the 453 still living participants of the cardiovascular sub-study, 64% (*n* = 290) agreed to participate in a re-examination included in the current study. We excluded individuals who did not undergo ambulatory BP monitoring (*n* = 12), were under 55 years of age (*n* = 112), did not work (*n* = 102), had insufficient number of valid BP measurements (*n* = 1, see above for definition) or had missing data on covariates (*n* = 4), resulting in a final sample of 59 participants. In all, we used a pooled sample of 208 participants from FIREA and DILGOM for our analyses. Regarding the FIREA participants, the excluded participants were significantly less physically active (39% vs. 26%, *p* = 0.001), more often smokers (11% vs. 4%, *p* = 0.005) and had greater BMI (26.8 vs. 26.0, *p* = 0.048). With respect to age, BMI or gender we found no significant difference. DILGOM participants did not diverge from excluded participants regarding age, BMI, gender, physical activity or smoking.

### Ambulatory BP

In the FIREA and DILGOM studies, a single 24-h ambulatory BP was performed with a Microlife WatchBP O3 monitor. BP was measured in FIREA during the work-days and in DILGOM during the weekdays from the non-dominant arm of the participant. The starting and ending times were arbitrary, but during normal working hours. Approximately, 1 min before each BP measurement, the device gave an alarm sound and the participant was then instructed to stop walking and preferably sit down with a relaxed arm. If the measurement failed, it was repeated immediately until the recording was successful. In FIREA, the BP was recorded every 30 min during the entire day; whereas in DILGOM, the recording was made every 20 min from 7 a.m. to 10 p.m. and every 30 min from 10 p.m. to 7 a.m. Participants reported times of going to bed and waking up, which was used to calculate average awake and asleep BP. Nocturnal BP dipping was calculated as [1 − (asleep BP/awake BP)] × 100 and expressed in percentages. Non-dipping was defined as a nocturnal BP reduction of < 10% (Parati et al. 2014).

### Work-related factors

Job strain was measured with an abbreviated version of Job Content Questionnaire in both cohorts (Karasek et al. 1998). The following job demands items were used: (1) “My job requires working very hard”, (2) “I am not asked to do an excessive amount of work” and (3) “I have enough time to get my job done”. Following job control items were used: (1) “My job allows me to make a lot of decisions on my own”, (2) “On my job, I am given a lot of freedom to decide how I do my work” and (3) “I have a lot of control over what happens at my job”. All items had a 5-point response scale, ranging from “I completely agree” to “I completely disagree.” The participants were categorized into high vs. low demands and high vs. low control based on median scores in the sample (FIREA: 3 for demands and 3.7 for control; DILGOM: 2.7 for demands and 4 for control).

Occupational status was obtained from the Finnish Pension Insurance Institute for the Municipal Sector in FIREA and from a questionnaire in DILGOM. Occupational status was categorized as manual (e.g., cleaner or maintenance worker) or non-manual (e.g., office or service occupations). Participants’ work-time mode was obtained by asking the participants whether they perform regular day work or shift work (including two- or three-shift work, evening or night work, unregular work).

### Covariates

Age and gender were obtained from the questionnaire. We also included cohort as a covariate. Leisure-time physical activity was divided into low and high groups based on the self-reported and pre-defined criteria in FIREA (< 14 metabolic equivalent) (Leskinen et al. 2018b) and DILGOM (< 4 h activity per week) (Borodulin et al. 2016). Body mass index (BMI) was calculated from measured weight and height in both cohorts and calculated as weight divided by height squared (kg/m^2^) (“Obesity: preventing and managing the global epidemic. Report of a WHO consultation” 2000). Smoking status was categorized as non-smokers (never and former) and current smokers. Participants self-reported their antihypertensive medication use.

### Statistical analyses

Characteristics of the participants are shown as mean values and standard deviation (SD) for continuous variables and proportions for categorical variables. Adjusted mean awake and asleep BP values and the nocturnal BP dipping percentage were calculated using the SAS GLM procedure. Differences between groups were compared using analysis of variance and the results are presented as mean and 95% confidence intervals (CI). Age, gender, BMI, antihypertensive medication, physical activity, smoking and cohort were included in all analyses as covariates. In addition, we performed sensitivity analyses regarding the association of work-related factors and nocturnal BP dipping, including only the participants who did not use antihypertensive medication. Statistical analysis was performed with SAS software, version 9.4 (SAS Institute Inc., Cary, North Carolina, USA).

## Results

Characteristics of the study participants are shown in Table 1. The majority of participants were women (75%) and mean age was 62.2 ± 2.8 years. The mean awake BP was 128.7/77.8 mmHg, while the mean asleep BP was 110.8/63.9 mmHg. 50% of the participants had awake hypertension and 44% asleep hypertension. 21% were categorized as systolic non-dippers and 11% as diastolic non-dippers.

**Table 1:** Characteristics of the study participants (*N* = 208)

	Total (*N* = 208)				FIREA (*N* = 149)				DILGOM (*N* = 59)			
	*N*	%	Mean	SD	*N*	%	Mean	SD	*N*	%	Mean	SD
Women, %	157	75			126	85			31	53		
Age, mean (SD)			62.2	2.8			62.4	0.9			61.9	5.1
Marital status: married	120	58			83	56			37	63		
Awake hypertension^a^	104	50			72	48			32	54		
Asleep hypertension^a^	91	44			63	42			28	47		
Awake SBP (mmHg)			128.7	12.1			128.8	12.0			128.6	12.4
Asleep SBP (mmHg)			110.8	11.2			110.4	11.0			111.8	11.6
Awake DBP (mmHg)			77.8	7.2			77.7	7.3			78.0	7.2
Asleep DBP (mmHg)			63.9	6.6			63.5	6.5			64.9	6.9
Nocturnal SBP dippers	165	79			120	81			45	76		
Nocturnal DBP dippers	185	89			137	92			48	81		
Blood pressure medication	52	25			37	25			15	25		
Low demands	101	49			70	47			31	53		
High control	110	53			89	60			21	36		
Occupational status: manual	45	22			36	24			9	15		
Shift work	66	32			35	23			31	53		
Low physical activity	47	23			38	26			9	15		
BMI (kg/m^2^)			26.5	4.5			26.1	4.4			27.4	4.5
Current smokers	12	6			7	5			5	8		

*SD* standard deviation, *SBP* systolic blood pressure, *DBP* diastolic blood pressure, *BMI* body mass index

^a^Awake blood pressure ≥ 135/85 mmHg or asleep blood pressure ≥ 120/70 mmHg

In our study, shift workers had significantly higher awake systolic BP than regular day workers, 136.5 mmHg (95% CI 131.9–141.1) vs. 132.5 mmHg (95% CI 128.3–136.6). The diastolic awake or asleep BPs did not differ significantly between these groups. We found no difference in awake or asleep BP values between manual and non-manual workers, workers with high and low job demands, or workers with high and low job control (Table 2).

**Table 2:** Mean awake and asleep blood pressures in categories by work-related factors (*N* = 208)

Work-related factor	Awake BP				Asleep BP			
	SBP (95% CI)	*p* value	DBP (95% CI)	*p* value	SBP (95% CI)	*p* value	DBP (95% CI)	*p* value
Occupational status		0.66		0.99		0.91		0.87
Non-manual (*n* = 163)	134.1 (130.0–138.3)		81.2 (78.8–83.7)		113.5 (109.6–117.4)		66.5 (64.2–68.8)	
Manual (*n* = 45)	133.2 (128.3–138.2)		81.2 (78.3–84.1)		113.7 (109.1–118.3)		66.7 (64.0–69.4)	
Work-time mode		0.03		0.15		0.37		0.79
Regular day work (*n* = 142)	132.5 (128.3–136.6)		80.7 (78.2–83.1)		113.0 (109.1–116.9)		66.7 (64.3–69.0)	
Shift work (*n* = 66)	136.5 (131.9–141.1)		82.3 (79.5–85.0)		114.6 (110.2–118.9)		66.4 (63.8–68.9)	
Job demands		0.29		0.34		0.82		0.65
Low (*n* = 101)	134.7 (130.4–139.0)		81.7 (79.1–84.2)		113.4 (109.4–117.4)		66.4 (64.0–68.7)	
High (*n* = 107)	132.9 (128.6–137.3)		80.7 (78.1–83.3)		113.7 (109.6–117.8)		66.8 (64.4–69.2)	
Job control		0.93		0.29		0.84		0.66
Low (*n* = 98)	133.9 (129.7–138.1)		81.6 (79.2–84.1)		113.4 (109.5–117.4)		66.7 (64.4–69.0)	
High (*n* = 110)	133.8 (129.3–138.3)		80.6 (77.9–83.2)		113.7 (109.6–117.9)		66.3 (63.8–68.8)	

Means are adjusted for age, gender, BMI, antihypertensive medication, physical activity, smoking and cohort. *p* values are for the differences between categories

*BP* blood pressure, *SBP* systolic blood pressure, *DBP* diastolic blood pressure, *CI* confidence interval

Table 3 shows the nocturnal BP dipping in categories by work-related factors. Diastolic nocturnal BP dipping was significantly greater in shift workers than in regular day workers (19% vs. 17%, *p* = 0.03). Systolic nocturnal BP dipping was significantly lower in participants with high job demands than in those with low job demands (14% vs. 16%, *p* = 0.04). In contrast, we did not find any significant associations between occupational status, job control, and nocturnal BP dipping values.

**Table 3:** Mean nocturnal blood pressure dipping (%) in categories by work-related factors (*N* = 208)

	Mean SBP dipping %	95% CI	*p* value	Mean DBP dipping %	95% CI	*p* value
Occupational status			0.40			0.82
Non-manual (*n* = 163)	15.3	13.6–17.1		18.0	15.9–20.0	
Manual (*n* = 45)	14.6	12.5–16.7		17.7	15.3–20.2	
Work-time mode			0.08			0.03
Regular day work (*n* = 142)	14.7	12.9–16.4		17.2	15.2–19.2	
Shift work (*n* = 66)	16.1	14.1–18.0		19.2	16.9–21.5	
Job demands			0.04			0.05
Low (*n* = 101)	15.8	14.0–17.6		18.7	16.6–20.7	
High (*n* = 107)	14.4	12.5–16.2		17.0	14.9–19.2	
Job control			0.64			0.49
Low (*n* = 98)	15.3	13.5–17.1		18.1	16.1–20.2	
High (*n* = 110)	14.9	13.0–16.9		17.6	15.4–19.8	

Means are adjusted for age, gender, BMI, antihypertensive medication, physical activity, smoking and cohort. Nocturnal BP dipping status was calculated as [1 − (asleep BP/awake BP)] × 100. *p* values are for the differences between categories

*SBP* systolic blood pressure, *DBP* diastolic blood pressure, *CI* confidence interval

Table 4 shows the nocturnal BP dipping results from a sensitivity analysis after excluding 52 participants on antihypertensive medication. In these analyses, we observed no significant differences in BP dipping values between groups categorized by work-related factors.

**Table 4:** Mean nocturnal blood pressure dipping (%) in categories by work-related factors among participants without antihypertensive medication (*N* = 156)

Work-related factor	Mean SBP dipping %	95% CI	*p* value	Mean DBP dipping %	95% CI	*p* value
Occupational status			0.78			0.97
Non-manual (*n* = 122)	16.2	14.3–18.2		19.3	17.0–21.5	
Manual (*n* = 34)	16.0	13.6–18.3		19.3	16.6–22.0	
Work-time mode			0.30			0.17
Regular day work (*n* = 106)	15.7	13.7–17.8		18.7	16.3–21.0	
Shift work (*n* = 50)	16.7	14.5–18.8		20.0	17.6–22.5	
Job demands			0.20			0.34
Low (*n* = 82)	16.5	14.6–18.5		19.6	17.3–21.8	
High (*n* = 74)	15.5	13.3–17.6		18.7	16.2–21.2	
Job control			0.77			0.85
Low (*n* = 73)	16.0	14.0–18.1		19.2	16.9–21.5	
High (*n* = 83)	16.3	14.2–18.4		19.4	17.0–21.8	

Means are adjusted for age, gender, BMI, physical activity, smoking and cohort. Nocturnal BP dipping status was calculated as [1 − (asleep BP/awake BP)] × 100. *p* values are for the differences between categories

*SBP* systolic blood pressure, *DBP* diastolic blood pressure, *CI* confidence interval

## Discussion

In this study, we examined the association of occupational status, work-time mode, job demands and job control with ambulatory BP among aging workers. We focused particularly on the relation of work-related factors and nocturnal BP dipping. We observed that individuals who performed shift work had significantly greater systolic awake BP and diastolic BP dipping than regular day-time workers. In addition, participants with high job demands had significantly lower systolic BP dipping than participants with low job demands. Job control and occupational status did not differentiate ambulatory BP profiles in aging workers.

To our knowledge, this is one of the first studies to study BP dipping among aging workers in this scale. We observed that shift workers demonstrated a significantly greater awake systolic BP and diastolic BP dipping compared to regular day-time workers. Unlike in our study, shift work has been related to an unfavorable dipping profile in three of four previous studies, (Fialho et al. 2006; Kitamura et al. 2002; Lo et al. 2008; Yamasaki et al. 1998). However, these studies included mainly younger individuals and only one study was performed using a single 24-h BP monitoring (Yamasaki et al. 1998), whereas the others examined the dipping profile changes in participants working in rotating shifts. In the study that was performed on middle-aged nurses untreated for hypertension, participants working in the evening or night shifts were more often systolic BP non-dippers than day-shift workers (Yamasaki et al. 1998). This difference was explained mainly by the higher systolic sleep BP. In our study, the differences in BP dipping values were explained mainly by the shift workers’ higher awake BP. These differences between studies may be partly explained by occupational differences because the participants of the previously mentioned study were nurses whereas our participants had a wide array of occupations. One major difference is also that, in contrast to the study with a sample consisting of nurses, only few of shift workers performed night shifts in our study. It has been earlier reported that circadian misalignment is the mechanism underlying the higher BP in shift workers (Morris et al. 2017). In addition, shift workers sleep less than day workers, express higher anger-in scores and, therefore, have probably more sympathetic nerve activity (Ohira et al. 2000). Therefore, it is possible that shift workers in our study had all these features, but most importantly, most of them were sleeping in the night in contrast to night-shift workers. Possibly, our shift workers had increased day BP because of stress but the relaxing effect of sleep was adequate for them as they had no circadian misalignment. In addition, our participants were also older and some were using antihypertensive medication. The exclusion of the participants with antihypertensive medication improved the nocturnal BP dipping profiles in general and abolished the significant difference between the regular day-time workers and shift workers. This can be partly explained by reduced statistical power. In addition, this could also reflect the fact that antihypertensive medication usually affects day-time BP values more than night-time values due to the more common dosing of the medications in the morning (Mancia and Parati 2004).

Studies on the relation of job demands and BP profiles have been extremely limited. In our study, we observed that workers with high job demands demonstrated a significantly smaller nocturnal systolic BP dipping than workers with low job demands. No differences in awake or asleep BP values were observed between these two groups. Although decreased nocturnal dipping has been related to adverse cardiovascular risk in previous studies (Salles et al. 2016), the clinical significance of this finding in individuals with high job demands remains unclear and requires further study in a prospective setting.

One earlier study has reported that low job control is related to higher asleep systolic BP and decreased nocturnal systolic BP dipping in younger hypertensive men (Fan et al. 2013). In older workers, low control, measured by possibility for influence and development at work, has also been associated with higher asleep systolic BP, but not with systolic nocturnal BP dipping (Mc Carthy et al. 2014). In our study, the differences in awake and asleep BP values and systolic nocturnal BP dipping between the low and high control groups remained small, even after performing a sensitivity analysis without participants on hypertensive medication. Two prior studies have also examined the association between job strain and ambulatory BP (Brown et al. 2006; Landsbergis et al. 2003b) regarding the occupational status. These studies reported that particularly workers performing manual labor may be more exposed to high BP induced by job strain. Our findings on the relation between job strain and ambulatory BP in aging workers, however, were limited.

The strengths of our study include having a relatively large number of participants with numerous occupational backgrounds, compared to previous smaller studies. Most of the previous studies considering work-time mode were also often performed at single workplaces in which psychosocial exposures may have a limited variance. Moreover, we included people with and without hypertension. The results of our study are, therefore, more generalizable to the aging general population as older workers commonly use antihypertensive medication. The current study has also some limitations which warrant discussion. First, although aging workers have been studied less in this context, they are also a distinct group of employees who may be somewhat healthier than the general population. This is reflected by the relatively low mean awake BP of the study sample (129/78 mmHg). It is also important to note that the proportion of non-dippers in our study was low (systolic/diastolic: 21/11%), which could partly explain the lack of significant differences between the groups. Furthermore, the awake BP values used in our study were the means of the whole awake period which could result in smaller between-group differences compared with mean BPs that would have been calculated separately for work- and free-time. We used shortened version of the Job Content Questionnaire although it has been shown to correlate with the complete scales (Fransson et al. 2012). Moreover, only a few of shift workers were performing night shifts. Besides, further analysis on sleep quality during the ambulatory monitoring would have helped to elucidate the impact of sleep quality on ambulatory BP but these data are unavailable in both study cohorts. In addition, as a cross-sectional study, this study can not offer information on the causal association between work-related factors and ambulatory BP.

## Conclusions

We conclude that these data provide evidence that shift work and high job demands are associated with ambulatory BP profile. Although shift work was related to a higher awake BP in our study, this increased cardiovascular risk may be offset by the fact that these individuals demonstrated greater BP dipping. Furthermore, hypertensive individuals with high job demands might benefit from 24-h ambulatory BP measurement as our findings suggest that they may have an increased risk of being non-dippers. Future prospective studies are needed to understand causality between these factors.
